# A Multifunctional Polymeric Micelle for Targeted Delivery of Paclitaxel by the Inhibition of the P-Glycoprotein Transporters

**DOI:** 10.3390/nano11112858

**Published:** 2021-10-26

**Authors:** Sobia Razzaq, Aisha Rauf, Abida Raza, Sohail Akhtar, Tanveer A. Tabish, Mansur Abdullah Sandhu, Muhammad Zaman, Ibrahim M. Ibrahim, Gul Shahnaz, Abbas Rahdar, Ana M. Díez-Pascual

**Affiliations:** 1 Department of Pharmacy, Quaid-i-Azam University, Islamabad 45320, Pakistan; sobiarazzaq11@gmail.com (S.R.); maisha.rauf001@gmail.com (A.R.); 2 NILOP Nanomedicine Research Laboratories, National Institute of Lasers and Optronics College, PIEAS, Islamabad 45650, Pakistan; abida_rao@yahoo.com; 3 Department of Entomology, Faculty of Agriculture & Environment, Islamia University of Bahawalpur, Bahawalpur 63100, Pakistan; sohailakhter4all@yahoo.com; 4 Department of Materials and London Centre for Nanotechnology, Imperial College of London, London SW7 2AZ, UK; t.tabish@imperial.ac.uk; 5 Department of Veterinary Biomedical Sciences, Faculty of Veterinary and Animal Sciences, PMAS Arid Agriculture University, Rawalpindi 46300, Pakistan; mansoorsandhu@uaar.edu.pk; 6 Department of Pharmacy, University of Central Punjab, Lahore 54000, Pakistan; m.zaman2157@gmail.com; 7 Department of Pharmacology, Faculty of Medicine, King Abdulaziz University, Jeddah 21589, Saudi Arabia; imibrahim1@kau.edu.sa; 8 Department of Physics, University of Zabol, Zabol 98613-35856, Iran; 9 Universidad de Alcalá, Facultad de Ciencias, Departamento de Química Analítica, Química Física e Ingeniería Química, Ctra. Madrid-Barcelona, Km. 33.6, 28805 Alcalá de Henares, Madrid, Spain

**Keywords:** P-gP efflux, biodistribution, mucoadhesion, resistance, fluorescent micelles

## Abstract

P-glycoprotein (P-gP) efflux-mediated multidrug resistance is a fundamental aspect of chemotherapeutic failure in oncology. The current study aims to deliver paclitaxel (PTX) specifically at the target site with improved in vivo efficacy of poorly permeable PTX against solid tumors. Multifunctional polymeric micelles as targeted delivery have been devised for loading and release of PTX. Mucoadhesion, permeation enhancement, oral pharmacokinetics, biodistribution, and toxicological studies were carried out to fully elucidate the therapeutic outcomes of the polymeric micelles. Ex vivo permeation studies indicated a 7.89-fold enhancement in the permeation of PTX with mucopermeating papain functionalized thiolated redox micelles (PT-R-Ms) compared to the pure PTX. Moreover, PT-R-Ms exhibited a higher percentage of apoptotic cells (42.9 ± 0.07%) compared to pure PTX. Biodistribution studies revealed that fluorotagged PT-RMs accumulated in excised tumors and organs. The higher fluorescence intensity indicated the mucopermeation of micelles across the intestine. The orally administered PT-R-Ms efficiently overcome intestinal barriers and inhibit the P-gP efflux pump, resulting in increased bioavailability of PTX (up to 8-fold) in comparison to pure PTX. The enhanced anti-tumor efficacy and reduced toxic effects are key aspects of efficient cancer therapy. This study demonstrates that the use of mucopermeating PT-R-Ms is an encouraging approach to overwhelm the permeation barrier in cancer treatment.

## 1. Introduction

Over the past few decades, extensive efforts have been made to solve the crucial issues of poor availability of chemotherapeutic drugs to the target site [1]; however, chemotherapeutic drugs encounter a wide variety of biological barriers, drug resistance, and patient non-compliance due to the availability of parental dosage form, lack of oral bioavailability, as well as side effects thereby leading to an inefficient therapeutic index [2]. The utmost occurrence of solid tumors is a leading cause of mortality in the 21st century. The therapy limitation in battling against solid tumors is P-glycoprotein (P-gP)-mediated-resistance and minimal diffusion of anticancer drugs inside cancer cells due to high activity of proteases enzymes [3]. Moreover, the high activity of enzymes leads to extensive degradation of the extracellular matrix (ECM). Overexpression of P-gP in tumor cells is mainly responsible for the efflux of diffused intracellular anticancer drugs, resulting in poor bioavailability of the drugs. Most of the anticancer drugs act as a P-gP substrate that binds to transporters and reduces their permeability due to efflux out of the drug via an adenosine triphosphate (ATP)-dependent process and reduced oral bioavailability [4]. P-gP is the most widely studied and potentially the most important efflux pump. It has been reported that P-gP is constitutively expressed in tumors as well as on the apical membrane of enterocytes, acting as a transmembrane transporter protein to lessen drug absorption through the small intestinal mucosa [5]. Thus, this protein may impair the therapeutic efficacy of chemotherapeutic drugs and affect their bioavailability following oral administration [6]. Mucus and ECM make crosslinked mesh and restrict the diffusion of drugs at the target site [7]. Another leading limitation of the current therapeutic regimen is the non-specific delivery of drugs that exposes normal organs to a high concentration of drugs resulting in undesirable side effects [8]. Taken together, all these restrictions for anticancer therapy offer a sturdy pace to the drug delivery system [9,10]. Targeted drug delivery systems can efficiently target the cellular/physiological barriers and can enable drugs to diffuse through mucus and ECM for the inhibition of P-gP efflux pump [11].

Several strategies have recently emerged to overcome the abovementioned crucial barriers, such as nanoengineered mucopermeating drug delivery systems [12]. These delivery systems can remarkably achieve the overriding goals (such as overcoming biological barriers, specific targeted delivery, P-gP efflux pump inhibition, accumulation in the pathological organs, receptor-intervened endocytic pathways, and so forth) that are likely intolerable with conventional treatment modalities [13]. A wide variety of polymeric excipients, including thiomers, pluronic, polysaccharides, and polyethylene glycol (PEG) derivatives, have been exploited as potent P-gP inhibitors [14]. Thiomers have attracted a lot of attention owing to their prominent efflux pump inhibitory properties, mucoadhesion, and permeation enhancement [15]. Amongst thiomers, anionic thiolated polymers have an excellent tendency to inhibit P-gP pumps and also show strong permeation ability. Limited efficacy of anticancer drugs/approaches makes it challenging to target efflux pump. To avoid the efflux of anticancer drugs out of the cells, paclitaxel (PTX) can be encapsulated into thiol-immobilized polymer micelles, which increase drug accumulation in the tumors.

Recently, our research group reported the novel PTX encapsulated papain functionalized thiolated redox micellar system against solid tumors [16]. Our previous in vitro results demonstrated the mucopermeating potential of PT-R-Ms. These micelles showed the highest internalization in tumor tissues, as revealed by time-dependent fluorescence microscopy. The diffusion/penetration, redox sensitivity, cytotoxic potential, ex vivo intestinal loop experiments, ECM degradation, tumor penetration, and tumor volume reduction were explored in our previous paper.

The enhanced mucopermeation ability facilitates the PT-R-Ms to diffuse/penetrate deeply across the intestinal mucolytic barrier via mucolytic enzymes such as papain. This provides PT-R-Ms specific targeting delivery and reduces toxicity towards normal cells [17]. The microtubule interfering agent PTX is the most effective chemotherapeutic agent against a broad range of cancers, such as colorectal, breast, lung, and ovarian. Given that PTX belongs to BCS class IV, it exhibits poor aqueous solubility and its intestinal uptake is severely hampered by P-gP drug efflux transporter, which limits its oral bioavailability [18]. Numerous P-gP inhibitors have been developed, but due to their systemic side effects, alternative ways to the established polymeric systems are sought. To improve the oral permeation of PTX as a first-line treatment drug for solid tumors across intestinal barriers and promote its internalization inside cancer cells, PTX has been encapsulated into the hydrophobic core of PT-R-Ms. The current study aims to further evaluate the potential of novel papain functionalized thiolated redox micelles (PT-R-Ms) for mucoadhesion, swelling, permeation, and P-gP efflux pump inhibition. It also examines the mucopenetration ability of PT-R-Ms by in vivo tracking. Moreover, the bioavailability, biodistribution, and toxicity of PTX encapsulated in Ms, R-Ms, PR-Ms, and PT-R-Ms were assessed.

## 2. Materials and Methods

Hyaluronic acid (HA), 1-ethyl-3-(3-dimethylaminopropyl) carbodiimide (EDAC), hydroxylamine, hydrogen peroxide, papain, pluronic (F407), lithocholic acid (LCA), disodium dihydrogen phosphate, sodium dihydrogen phosphate, glucose, sodium chloride, sodium borohydride, potassium chloride, magnesium chloride, fetal bovine serum (FBS), penicillin, streptomycin, Nile red, and dialysis membrane (cut-off value 12KD) were purchased from Sigma-Aldrich (Baden, Germany). PTX was kindly supplied by NovaMed Pharmaceuticals Pvt. Ltd. (Lahore, Pakistan). All the solvents used were of analytical and high-performance liquid chromatography (HPLC) grade.

### 2.1. Synthesis and Characterization of Micelles

The synthesis of PTX encapsulated PT-R-Ms was carried out as reported earlier [16]. Briefly, the PT-R-Ms were prepared from papain grafted thiolated hyaluronic acid-pluronic F127 disulfide-linked lithocholic acid (Pap-THA-F127-SS-LCA) via probe sonication method. The polymer conjugate (10 mg) was hydrated into water (5 mL) and stirred for 20 min. Subsequently, PTX (6 mg) was dissolved in ethanol and added dropwise to the polymeric solution. Then, the conjugate was placed under probe sonication in pulse mode (12 s on/6 s off). The resulting solution was filtered through a 0.45 µm filter, lyophilized, and stored at 4 °C. The average particle size (nm), zeta potential (mV), and polydispersity index (PDI) of the synthesized micelles were measured with a NanoSizer (Malvern Instruments, Malvern, UK). Morphological studies were conducted by transmission electron microscopy (TEM) using a Nova Nano TEM 450 instrument (FEI Technologies Inc., Hillsboro, OR, USA). The samples were prepared by placing 1 mg/mL nanomicelle solution onto copper grids before being stained with 2% phophotungstic acid. The drug loading (DL%) and the encapsulation efficiency (EE%) were calculated according to the following formula:

(1) $$\;\mathrm{EE}\;\left(\%\right)=\frac{\mathrm{Amount}\;\mathrm{of}\;\mathrm{drug}\;\mathrm{in}\;\mathrm{micelles}}{\mathrm{Total}\;\mathrm{amount}\;\mathrm{of}\;\mathrm{feeding}\;\mathrm{drug}\;}\times 100\%$$

(2) $$\;\mathrm{DL}\;(\%)=\frac{\mathrm{Amount}\;\mathrm{of}\;\mathrm{drug}\;\mathrm{in}\;\mathrm{miclles}}{\mathrm{amount}\;\mathrm{of}\;\mathrm{feeding}\;\mathrm{polymer}\;\mathrm{and}\;\mathrm{drug}}\times 100\%$$

Glutathione (GSH)-triggered release was investigated in vitro from Ms, R-Ms, PR-Ms, and PT-R-Ms formulations (without GSH) and from PT-R-Ms in the presence of 20 mM GSH.

### 2.2. Swelling Index and Rheological Study

The swelling behavior of the micelles was investigated by the gravimetric method [19]. Initially, pre-weighed compressed tablets of non-redox micelles (Ms), redox micelles (RMs), papain functionalized redox micelles (P-RMs), and papain functionalized thiolated redox micelles (PT-R-Ms) were attached to the needle of the syringe placed in a beaker containing phosphate buffer (pH 6.8). At regular time intervals, the discs were removed from the buffer medium and accurately weighed. The weight gain was calculated as a function of time by using the equation:

(3) $$\mathrm{Swelling}\;\mathrm{index}\;(\%)=\frac{\mathrm{Wt}-\;W0\;}{W0}\times 100\%$$

where W_0_ is the initial weight of the disc in dry state and W_t_ represents the wet weight of the swollen disc at time t.

The viscoelastic properties of Ms, R.Ms, P-RMs, and PT-R-Ms were evaluated using a cone plate viscometer (CE AMETEK Brookfield, assembled in Middleborough, MA, USA, with Volt/Freq: 90-260 V-50/60 Hz and a power of 150 VA). Mucin extracted from bovine stomach (5 g) was mixed with 1000 mL phosphate buffer (0.1 M, pH 7.4), and the final pH was adjusted to 6.8. Afterward, 1 mL of the redox micelles (R-Ms, P-R-Ms, PT-R-Ms) or non-redox micelles (Ms) was added to 6 mL of the extracted mucin and incubated at 37 °C in a water bath for 4 h. At predefined intervals of 0, 2, and 4 h, the viscoelastic properties were measured by adding 500 µL of the mucin–micelle mixture to the viscometer [20]. The rheological synergism parameter (Δη) was calculated at a shear rate of 60 s^−1^ following the equation:

Δη = η_mix_ − η_muc_ (4)

where η_mix_ is the apparent viscosity of the mucin–micelle mixture (Pas), while η_muc_ is the apparent viscosity of a mucin dispersion with the same concentration to that of the mixture (Pas).

### 2.3. Enhanced Intracellular Accumulation by Inhibiting P-gP Efflux

Ex vivo permeation enhancement was analyzed using the everted sac method [21] by comparing the synthesized formulations with PTX dispersion. Briefly, the study was conducted on the intestine of healthy rats weighing between 250–300 g. The rats were sacrificed and the abdomen was opened with a middle incision. The intestine was immediately removed, thoroughly washed with Krebs ringer solution (pH 6.5), and cut into pieces of 4–5 cm. The intestine was everted by carefully passing a narrow glass rod from one end of the intestine and then gently rolling it on a glass rod. All the pieces were stored in an oxygenated Krebs ringer solution at 4 °C until further use. A solution of 1% Tween-80 was added to enhance the wettability of PTX. Each segment was tied at one end with a silk suture, and 1 mL of sample (1 mg/mL) was carefully filled in the sac using a hypodermic syringe and the other end was tied with a silk suture. To compare the apparent permeation enhancement with micelles, verapamil (100 μg/mL), a P-gP inhibitor, was filled in one sac. All filled sacs were immersed in tubes filled with 10 mL of oxygenated Krebs solution and incubated at 37 °C under gentle mechanical shaking. The samples were collected at predefined times from the surrounding medium and replaced with the same amount of fresh solution. The samples were analyzed using an HPLC method developed and validated according to the International Council for Harmonization (ICH) guidelines for the quantification of PTX. The method was established using an LC-20 HPLC system (Schimadzu, Japan) equipped with an SPD-20A UV detector. The mobile phase consists of phosphate buffer (pH 7.2) and acetonitrile (68:32). A C18 (250 cm × 4.6 mm; 5 µm packing L1) column was used, and the temperature was set at 40 °C. The injection volume utilized for the sample analysis was 20 µL at a flow rate of 1 mL/min, and it was analyzed at 227 nm. The method was validated in terms of accuracy, precision, robustness, specificity, limit of quantification (LOQ), and the limit of detection (LOD). The apparent permeability was calculated using the following equation:

Apparent permeability (µg/cm^2^) = (concentration × volume)/mucosal surface area

The mucosal surface area was calculated assuming that the intestine is a cylinder by using the formula:

Mucosal surface area (cm^2^) = circumference (π × diameter) × length

### 2.4. Biocompatibility against Human Macrophages

Biocompatibility studies of pure PTX, Ms, RMs, PR-Ms, and PT-R-Ms were performed against human macrophages. Ficoll-percoll gradient technique was used to isolate human macrophages. Ficoll solution was prepared by dissolving 5.6 g of ficoll in 9.5 mL deionized water and 5 mL gastrografin to achieve a density of 1.070 g/mL. The fresh human blood (10 cm^3^) was diluted three times with Hanks buffer salt solution (HBSS), layered onto a percoll gradient and then centrifuged at 400× *g* for 5 min to isolate the macrophage layer. The separated cells were suspended in RPMI medium (10% FBS, 100 U/mL penicillin, 0.1 mg/mL streptomycin, and 25 mM HEPES) and incubated at 5% CO_2_. Viable cells were seeded into 96 well plates and treated with 100, 50, 25, 15, 10, 5 µg/mL concentrations of pure PTX, Ms, RMs, PR-Ms, PT-R-Ms, and vehicle control cells. Normal cells were taken as negative controls, while cells treated with 1% Triton X were used as positive controls to investigate the viability. After 48 h incubation, IC50 was calculated using GraphPad Prism software (version 7.02) [22].

### 2.5. Cellular and Tumor Tissue Uptake

To analyze the internalization of curcumin (a P-gP inhibitor) into tumor cells, HCT-116 tumor cells were used. Curcumin as a fluorescent probe was encapsulated within the core of PT-R-Ms micelles following a similar procedure to that mentioned earlier for PTX loaded micelles. The HCT-116 cells were seeded into 6 well plates at a density of 1 × 10^6^ cells/well. After 24 h the cells became confluent and were incubated with curcumin-loaded PT-R-Ms for different time periods (0, 6, 12 h). Upon incubation, the medium was removed and the cells were washed with cold PBS. Subsequently, the uptake of curcumin was monitored by fluorescence microscopy (Evos^®^ FL Cell Imaging System, Thermo Fisher Scientific, Waltham, MA, USA) [23]. Ex vivo assays based on the tumor tissue slice model were used to analyze the curcumin uptake by inhibition of P-gP efflux pump overexpressed on tumor cells, as reported earlier [16]. Different slides of tumor tissue were prepared and stained with curcumin-loaded PT-R-Ms. Similarly, HCT-116 tumor tissue cells were incubated for different time intervals to monitor the uptake of curcumin.

### 2.6. Cell Apoptosis

The effect of redox micelles on HCT-116 cancer cells was evaluated by flow cytometry using different redox formulations (RMs, PR-Ms, PT-R-Ms) micelles with pure PTX. Briefly, HCT-116 cells at a density of 1 × 10^4^ cells/well were seeded in 6 well plates for 48 h with these formulations at a PTX concentration of 0.1 mg/mL. After treatment, the cells were harvested and washed twice with cold PBS. Then, 1 × 10^5^ cells were collected and resuspended with 300 mL of a binding solution, followed by the addition of 5 mL of FITC-Annexin V and 5 mL of propidium iodide (200 mg/mL), and subsequently incubated at 37 °C under dark conditions for 15 min. Then cell suspension was immediately analyzed by flow cytometry (BD FACSDIVA 9.0 version, BD Corporation, Franklin Lakes, NJ, USA) [24].

### 2.7. Tumor Reduction Study

Fresh tissues of colorectal cancer (CRC) were taken as surgical waste from patients that underwent surgical resection at Jinnah Hospital (Lahore, Pakistan). The investigation was officially approved by the local ethics committee (#BEC-FBS-QAU2019-200) and informed consent was achieved from patients prior to participation. Afterwards, tumor tissues were preserved on ice for further processing. Tumor chunks of equal size (thickness: 600 m, diameter: 5 mm) were cut by a Krumdieck microtome [25]. The tumor chunks were incubated under the optimal conditions for primary cancer cell growth (37 °C in a humidified 5% CO_2_ environment for 28 days). Afterwards, the tumor chunks were divided into two groups: one served as a control and the other treated with PTX loaded PT-R-Ms was used for comparison. The tumor volume reduction was examined after 0, 7, 14, and 28 days. Tumor size was measured using a Vernier caliper and tumor volume was calculated using the formula: V = 0.5 × a × b^2^, where ‘a’ and ‘b’ represent the major and minor diameter, respectively.

### 2.8. Bioimaging

#### 2.8.1. In Vivo Micelles Tracking within the Intestine

Mucopenetration of PT-R-Ms at different time intervals was investigated in mice small intestine. All animal experiments were performed by the standard protocol of the Bio-Ethical committee of Quaid-Azam University, Islamabad, Pakistan (#BEC-FBS-QAU2019-200), considering EU directives 2010/63 for animal studies. Healthy mice weighing 20–25 g were selected and kept in an animal house with free access to food and water before experimentation and were distributed into 3 groups (*n* = 3). Fluorotagged PT-R-Ms (100 μg/mL) were given to fasted male BALB/c mice via oral gavage. Mice were anesthetized with urethane solution, and the ileum was exposed from a small incision in the abdomen [26]. The anesthetized mice were examined under iBox Explorer2 (iBox^®^ Explorer2 Imaging Microscope; UVP Ltd., Cambridge, UK) after 6 and 12 h, and the fluorescence was observed as indicated by the penetration of micelles within the small intestine. Mucopenetration of fluorescent-loaded formulations was also confirmed via examination of the sliced intestinal sections.

#### 2.8.2. In Vivo Bioavailability of PTX at the Target Site

Healthy female rats weighing 120–150 g were selected and fasted overnight with free access to water and were randomly distributed into four groups and each group had four rats (*n* = 4). PTX loaded R-Ms, PR-Ms, PT-R-Ms, and pure dispersion of PTX at a dose of 20 mg/kg body weight were administered to the rats by oral gavage. Blood samples (approximately 1 mL) were collected from the tail vein at different intervals of 1, 4, 6, 8, 10, 12, 24, 48, 72, and 96 h in microcentrifuge tubes containing heparin. These blood samples were centrifuged at 3500 rpm for 10 min to separate the plasma, and the plasma samples were modified for HPLC to measure PTX levels [27].

### 2.9. In Vivo Biodistribution and Acute Toxicity

The fluorotagged PT-R-Ms were given orally to rats by oral gavage. After 48 h, rats were sacrificed and their major organs were excised to investigate the formulation distribution within them [28]. The fluorescence intensity of the excised organs (heart, liver, spleen, and kidney) was examined using the iBox Explorer2 (iBox^®^ Explorer2 Imaging Microscope; UVP Ltd. The system was set on a 535/45 nm excitation filter and a 605/50 nm emission filter with the automated BioLite™ MultiSpectral Light Source. Images were taken at 0.17× magnification, and the intensity was kept at 6 using the 3.2 MP OptiChemi 610 camera (Vision Works^®^ LS Acquisition, AnlyticJena, Jena, Germany). An analysis software was used to study the images.

Acute oral toxicity of RMs, PR-Ms, and PT-R-Ms was evaluated in rats for 14 days following OECD 425 guidelines. In vivo studies were preceded as per the approved guidelines of the BioEthical Committee of Quaid-i-Azam University (#BEC-FBS-QAU2019-200). Healthy, female rats weighing 150 g and aged 10–14 weeks were taken from the animal house. Rats were divided into 4 groups (*n* = 4) and kept under a standard condition of food and water in the controlled environment. Group 1 was taken as control and given normal saline, group 2 comprised the Ms, group 3 the PR-Ms, and group 4 the PT-RMs. The dose (20 mg/kg) was administered orally through oral gavage. The rats were kept under observation for 24 h for change in weight and visual observations for mortality, behavior pattern (fur and skin, consistency of feces, urination color, salivation, eyes, respiration, sleep pattern, mucous membrane, convulsions, and coma), physical appearance changes and signs of illness were monitored daily. After 14 days, the rats were sacrificed for blood and serum biochemistry and tissue histology studies [29].

### 2.10. Serum Biochemistry, Complete Blood Count, and Organ to Body Ratio Analysis

After 14 days, serum biochemistry was performed to check the toxicity induced by the Ms, RMs, PR-Ms, and PT-R-Ms. The blood from each rat was drawn through cardiac puncture into BD vacutainer blood collection tubes. The blood was centrifuged at 1200× *g* for 10 min to separate the plasma. The clear supernatant was carefully removed and stored at −20 °C. Liver function tests (LFTs) including alkaline phosphatase (ALP), serum glutamic-pyruvic transaminase (SGPT), serum glutamic-oxaloacetic transaminase (SGOT) and bilirubin, renal functions tests (RFTs) including urea and creatinine, serum electrolytes (Na, Mg, Ca, and P), glucose and total protein were analyzed using the serum.

Changes in the organs weight were measured to evaluate the toxicity of tested formulations. The vital organs (heart, kidneys, liver, and spleen) were removed from the rats after being sacrificed, washed with normal saline, and then weighed individually. The weights of organs from treated groups were compared with a control group and body mass index was calculated using the following formula [30]:

$$\mathrm{organ}-\mathrm{body}\;\mathrm{weight}\;\mathrm{index}\;(\%)=\frac{\mathrm{organ}\;\mathrm{weight}}{\mathrm{body}\;\mathrm{weight}}\times 100\%$$

### 2.11. Histopathology of Vital Organs and Tissue Distribution Analysis

Vital organs (liver, kidney, heart, and spleen) were examined for toxicity and to notice any pathological changes [31]. The organs were stored in 10% formalin solution. A paraffin section (0.5 µm) was cut into the same size as the organ via a rotary microtome and was properly fixed. Tissue sections were then fixed onto glass slides followed by staining with hematoxylin and eosin periodic acid Schiff (PAS). The sections were examined under a microscope for any toxic effect produced by the redox and non-redox micelles.

Tissue distribution of PTX loaded Ms, RMs, PRMs, and PT-RMs was investigated using tissue homogenate analysis. Briefly, a weighed amount of chopped organ (liver, kidneys, heart, and spleen) was mixed with 1 mL normal saline (0.9% *w*/*v*) and homogenized. 1 mL of mobile phase was added to extract the drug from tissues and the mixture was further sonicated for 15 min followed by centrifugation at 5000× *g* for 15 min. The supernatant was carefully separated and analyzed using the HPLC method previously developed for PTX quantification in plasma samples.

### 2.12. Statistical Analysis

All the results were obtained using a two-way analysis of variance (ANOVA) to compare the results of different treatments with Ms, RMs, PR-Ms, PT-R-Ms, and PTX. All the experiments were performed in triplicate. Data are given as mean ± SD. A *p*-value less than 0.05 (* *p* < 0.05) was considered to indicate the significant difference.

## 3. Results

### 3.1. Synthesis and Characterization of Micelles

**Figure 1:**
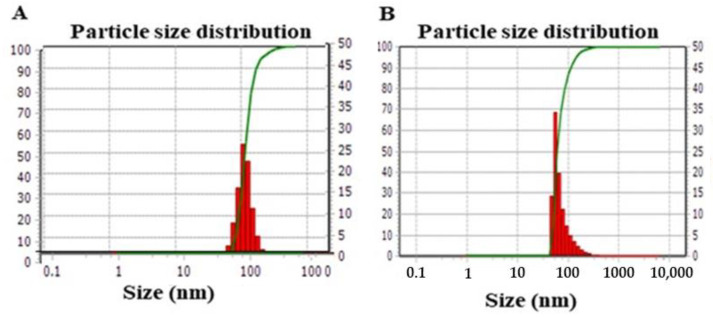
Gaussian particle size distribution obtained by DLS for (**A**) Ms and (**B**) PT-R-Ms. Data are expressed as mean ± SD (*n* = 3).

**Figure 2:**
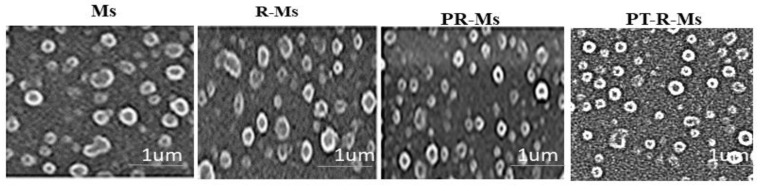
Representative TEM images of Ms, R-Ms, PR-Ms, and PT-R-Ms.

The mean particle size of the synthesized PT-R-Ms and the blank micelles (Ms) obtained by DLS was 90 ± 7 and 105 ± 16 nm, respectively, as shown in Figure 1. The details of the different formulations, including their nomenclature, chemical composition, synthesis method, size, drug loading (DL%), and encapsulation efficiency (EE%), are given in Table 1. Typical TEM images of the different formulations are shown in Figure 2.

**Figure 3:**
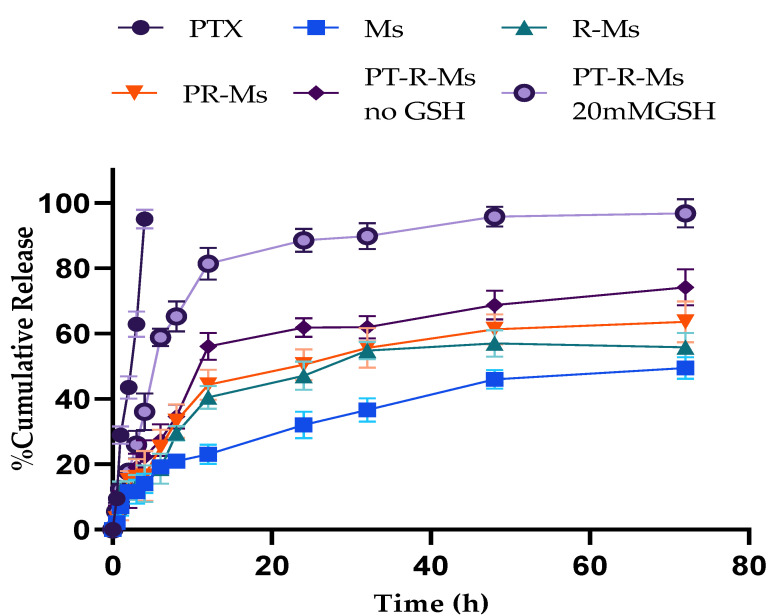
In vitro release of Ms, R-Ms, PR-Ms, and PT-RM (without GSH) and PT-RMs (20 mM GSH). Results are expressed as mean ± SD (*n* = 3).

Redox responsive multifunctional micelles remained indolent in the physiological environment and became activated when they reached a reductive environment, which is another approach for targeted site-specific delivery. As a proof-of-concept, glutathione (GSH)-triggered release (to mimic the redox tumor microenvironment) was investigated in vitro from Ms, R-Ms, PR-Ms, and PT-R-Ms formulations (without GSH) and from PT-R-Ms in the presence of 20 mM GSH. The results for the different formulations are shown in Figure 3. The controlled burst release was confirmed by applying kinetic models of drug release (zero-order, Higuchi, Korsmeyer–Peppas, and Hixon Crowell) to the different formulations, and the results are gathered in Table 2. The Korsmeyer–Peppas model was proven to be suitable to explain the release kinetics from the PT-R-Ms, which is a prerequisite for polymeric systems.

### 3.2. Swelling Study

**Figure 4:**
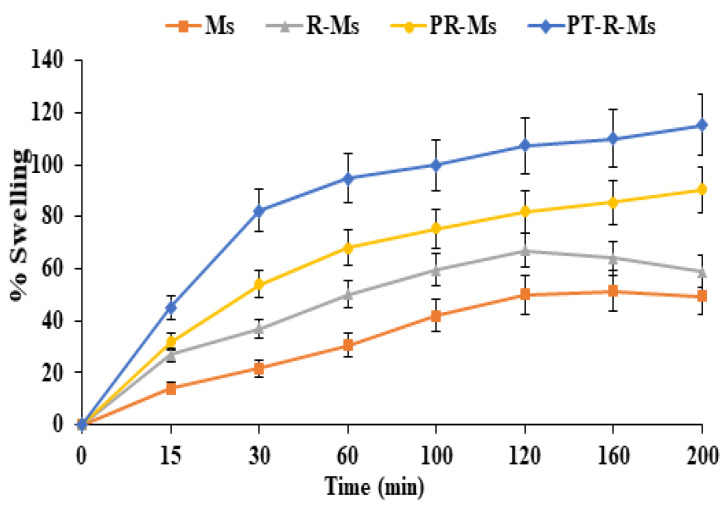
Swelling studies of Ms, R-Ms, PR-Ms, and PT-R-Ms. Experiments were carried out for 3 h in phosphate buffer (pH 7.4, 0.1 M). Results are expressed as mean ± SD, *n* = 3, *p* < 0.05.

Appropriate swelling is a prerequisite for thiomeric nanocargoes [32]. The higher swelling capability of thiolated polymers (thiomers) compared to unfunctionalized ones arises from their reduced crosslinking density and weaker interactions among the polymeric network, which enable them to uptake a larger amount of water. The water uptake capacity of the as-synthesized mucopermeating polymer was investigated. Time-dependent swelling behavior was observed for a flat tablet of non-redox micelles (Ms) and redox micelles (R-Ms, P-RMs, PT-R-Ms) that became hydrated and gained weight upon incubation in PBS (pH 6.8) before the polymer disruption [33]. Disulfide bond containing mucopermeating polymer exhibited greater cohesiveness, consequently accelerating the swelling of the polymer chains and the retention time. PT-R-Ms exhibit higher water absorption capacity than Ms, RMs, and PR-Ms. PT-R-Ms hydrate in 150 min while the hydration process in Ms, RMs, and PR-Ms took less than 60 min. The enhanced mucus hydrogel penetration significantly increases the residence time of the micelles within the mucus membrane. Disulfide containing micelles (PT-R-Ms) absorb more water molecules and exhibit improved disintegration, as shown in Figure 4. Furthermore, decelerated water uptake can be observed for the non-redox micelles that lack disulfide bonds.

### 3.3. Mucoadhesive Behavior via Rheological Synergism

**Figure 5:**
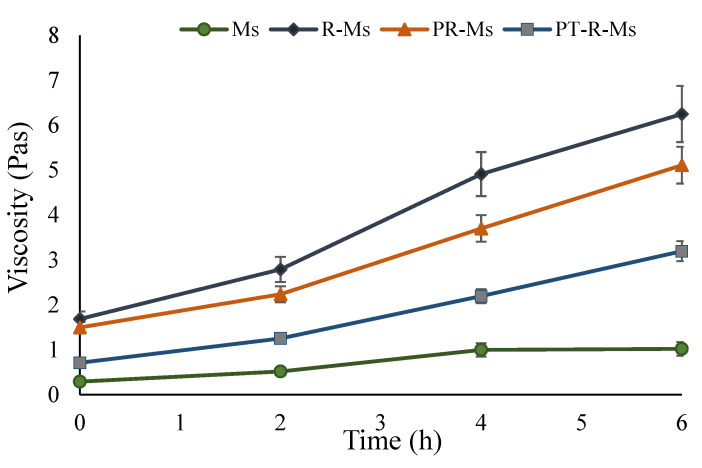
Rheology studies of Ms, R-Ms, P-R-Ms, and PT-R-Ms. Results are expressed as mean ± SD, (*n* = 3), *p* < 0.05.

A suitable strategy to overcome the intestinal mucus hydrogel barrier is the utilization of a mucolytic/proteolytic enzyme that can cleave the muco-glycoprotein. To evaluate and analyze the mucus integrity, a rheological study was performed that determined the time-dependent viscosity (Figure 5). The mucus degradation by the proteolytic enzyme decreases the viscoelastic parameters, which can be correlated with the proteolytic ability of the enzyme immobilized over the polymeric micelles. The dynamic properties of disulfide-containing mucopermeating polymer were assessed by measuring the elastic modulus G’ and the viscous modulus G″. The rheological parameter (Δη) is directly related to the mucoadhesion of the mucin. It has been suggested that the rheological synergism between a polymer and mucin indicates the extent of mucoadhesive bond strength [34]. A 1.4-fold increase in the viscosity of the PT-R-Ms was found after 6 h interaction with mucin, which demonstrates enhanced mucoadhesion, ascribed to the presence of covalent interactions between disulfide bonds between cysteine residues of the polymer and mucin glycoprotein. The increase in viscosity with increasing time of interaction with mucin is due to the absorption of water from the surrounding media. Results shown in Figure 5 demonstrate that PT-RMs viscosity lies between that of R-Ms and PR-Ms, since they comprise a large number of disulfide bridges, while the viscosity of Ms increases only marginally due to the absence of cysteine residues. The decelerated mucoadhesive behavior of PT-R-Ms might be due to the presence of a papain enzyme that has a mucopermeating ability to cleave the thick bonds of the glycoprotein.

### 3.4. Enhanced Intracellular Accumulation by Inhibiting P-gP Efflux

**Figure 6:**
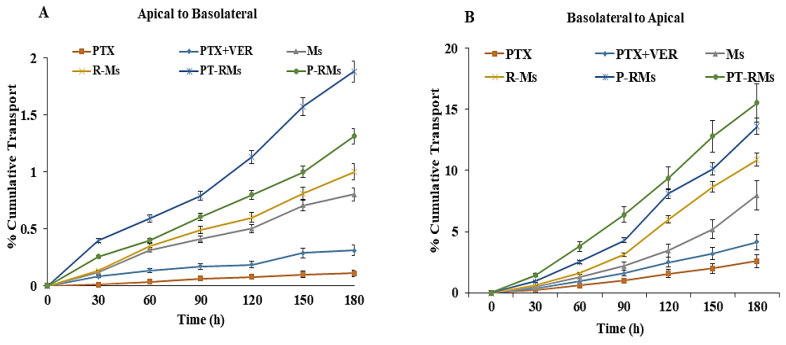
Ex vivo studies of PTX alone, PTX with verapamil, Ms, R-Ms, P-R-Ms, and PT-R-Ms across rat intestine: (**A**) Apical to basolateral permeation studies; (**B**) basolateral to apical permeation studies. PTX transport is expressed as cumulative transport. Results are given as mean ± SD (*n* = 3) (*p* < 0.05).

Besides uptake, most anticancer drugs are effluxed out from the enterocytes due to the presence of efflux transporters on the surface of the intestinal mucosa [35]. Amongst these transporters, P-gP deserves special attention since it promotes the release of several drugs and reduces their absorption and bioavailability. PTX can suffer from limited permeation in the presence of P-gP transporter given that it acts as its substrate; however, when it is encapsulated within micelles, the nanocarriers associate with the particles, hence it is not expected to be a substrate for efflux pump [36]. PTX permeation in the presence and the absence of verapamil (P-gP inhibitor) on everted rat intestinal sacs is plotted in Figure 6A. Results reveal that in the presence of the P-gP inhibitor (verapamil 100 μg/mL), PTX absorption into the sac contents increased significantly, by 1.82-folds (*p* < 0.05) as compared to the control. The significant absorption enhancement of PTX is achieved via encapsulation into the micelles. The apparent permeability coefficient (Papp) enhancement ratio was 1.98-, 5.78-, 6.21- and 7.89-fold higher for Ms, R-Ms, P-R-Ms and PT-R-Ms formulations, respectively, as shown in Table 3. Certain efflux, including P-gP and influx pumps, are present in the epithelial cells of the intestine. This P-gP efflux pump influences drug absorption and facilitates the efflux of PTX from the intestine. Furthermore, PTX, Ms, R-Ms, PR-Ms, and PT-RMs were evaluated for their Papp in the reverse basal to an apical direction (secretory transport), and the results are plotted in Figure 6B. Experimental data indicate that the secretory Papp of PTX across mucosa was 5.46-fold higher than the absorptive (apical to basal) Papp, indicative of the secretory orientation of PTX across the mucosa. PT-R-Ms trapped the PTX inside their core and diffused easily into the target site, and also inhibited the overexpressed P-gP transporter by altering its protein conformation and causing steric hindrance that limits the active efflux. In addition, thiolated hyaluronic acid is responsible for P-gP inhibition owed to the covalent interactions between the –SH groups of the thiolated polymer with the cysteine residues of P-gP transporters. By inhibition of P-gP transporters, PTX accumulated at a target site and improved its therapeutic potential and bioavailability at the target site.

### 3.5. Cellular and Tumor Tissue Uptake

**Figure 7:**
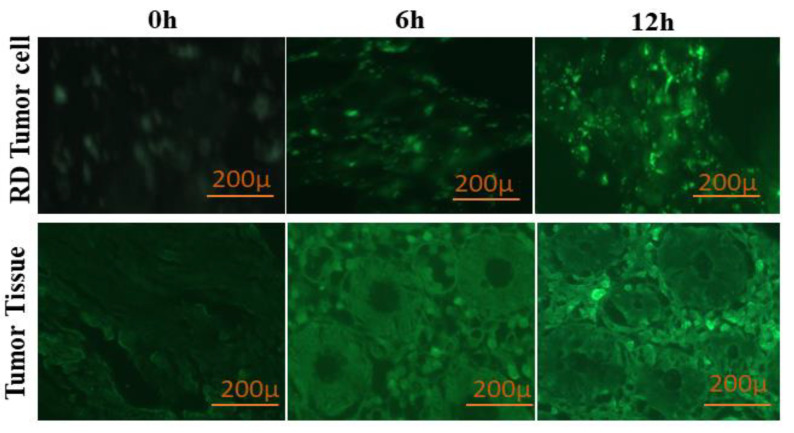
Internalization of curcumin (1.5 nM) in HCT-116 cells and tumor tissues for 0, 6, and 12 h by inhibition of the P-gP efflux pump.

Intracellular accumulation can be enhanced by inhibiting the P-gP efflux pump present on the tumor cells. To confirm this postulate, curcumin was loaded into PT-R-Ms micelles and incubated in HCT-116 cell culture medium and tumor tissue slides. The fluorescence was then measured by fluorescence microscopy (Figure 7). After 0 h, no fluorescence was observed, albeit, over time, it gradually increased owed to the curcumin uptake inside the cells. The maximum curcumin uptake was observed after 12 h, due to the inhibition of the P-gP efflux pump that enables the curcumin to accumulate inside the cells and emit fluorescence. A similar trend was found for tumor tissues: a drop in fluorescence intensity was observed after 6 h, while the fluorescence became more intense with increasing time, as clearly shown in Figure 7.

### 3.6. Biocompatibility against Human Macrophages

**Figure 8:**
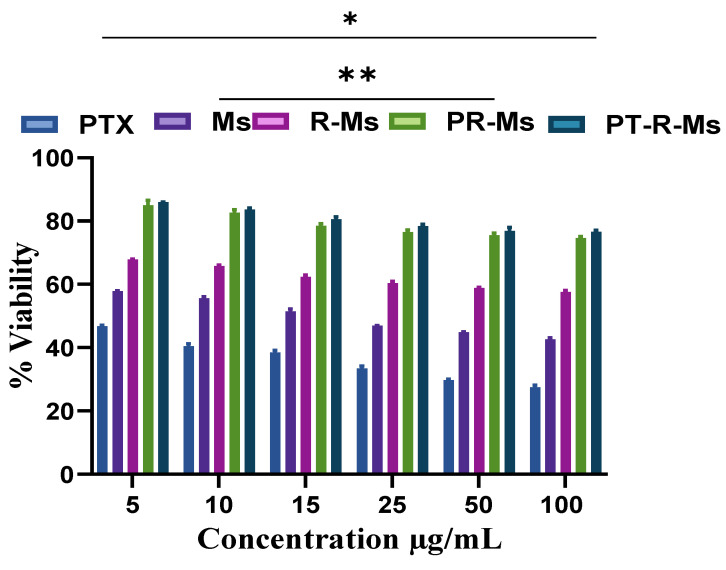
Biocompatibility analysis of Ms, R-Ms, P-R-Ms, and PT-R-Ms at different concentrations to evaluate the toxicity against macrophages isolated from fresh human blood via MTT assay. Results are shown as mean ± SD, (*n* = 3), *p* < 0.05 (*), *p* < 0.01 (**).

Nanocarriers exhibit different size, shape, surface charge, and hydrophilicity/hydrophobicity that influence the blood chemistry. Micelles with the highest surface potential and charge affinity are prone to circulate for a longer time through the blood flow, and show weaker interactions with macrophages. The prospective toxicity and biocompatibility of a pure PTX suspension, PTX loaded Ms, RMs, PR-Ms, and PT-RMs against human macrophages are shown in Figure 8. The results illustrate that high concentrations of the pure drug suspension are cytotoxic while micelles unveiled more than 80% viability at higher concentrations (5, 10, 15, 25, 50, 100 µg/mL).

### 3.7. Cell Apoptosis

**Figure 9:**
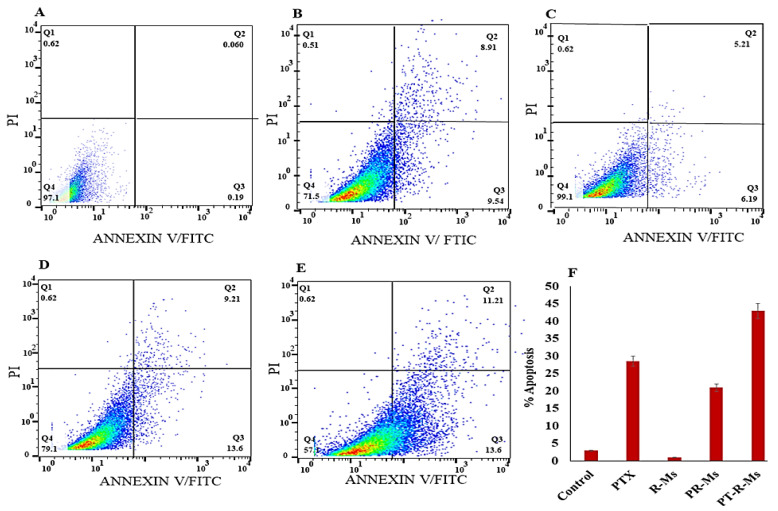
Apoptosis against HCT-116 cell lines induced by different formulations: (**A**) normal saline as control; (**B**) pure PTX; (**C**) R-Ms; (**D**) PR-Ms; (**E**) PT-R-Ms; (**F**) percentage of apoptosis in the pure drug and the different formulations. The results are obtained from three independent experiments. Data are shown as the mean ± SD (*n* = 3) (*p* < 0.05).

The induction of apoptosis mediated by PT-R-Ms against HCT-116 cell lines was analyzed by flow cytometry, and the results are shown in Figure 9. The percentage of apoptotic cells was 42.9 ± 0.07% in the PT-R-Ms, a value significantly higher than that found for pure PTX (28.57 ± 0.06%), R-Ms (0.950 ± 0.001%), and PR-Ms (20.09 ± 0.25%). The PT-R-Ms increased the apoptosis of HCT-116 cancer cells more effectively than the other formulations. Further, the results indicate that PT-R-Ms could induce more apoptosis than pure PTX.

HCT-116: human colorectal carcinoma cell line, R-Ms: redox micelles, P-RMs: papain functionalized redox micelles, P-RMs: papain functionalized thiolated redox micelles. Staining with Annexin V FITC and propidium iodide (PI).

### 3.8. Tumor Reduction Study

**Figure 10:**
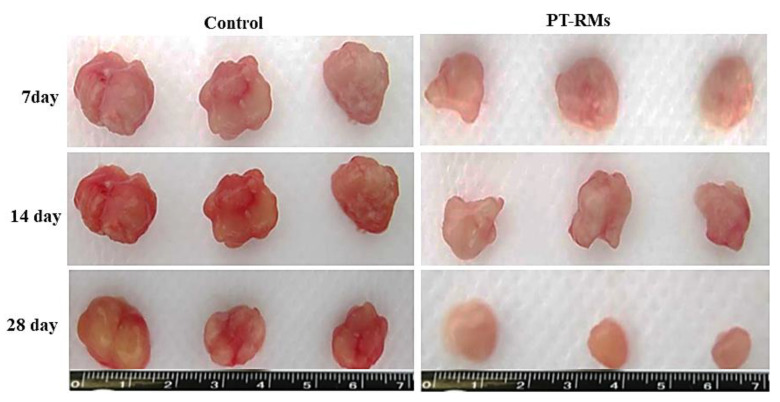
Time-dependent tumor volume reduction study. A volume reduction is found for freshly excised tumor chunks incubated with PTX loaded PT-R-Ms, while control tumors remained the same size (*p* < 0.05).

The time-dependent tumor volume reduction study was performed on freshly excised CRC tumors. The tumor chunks were incubated for 7, 14, 21, and 28 days, as illustrated in Figure 10. As can be observed, the volume of the living tumor chunks was reduced during the period of the study. The size of the control tumor chunk did not decrease, while those incubated with PTX encapsulated into PT-R-Ms showed reduced size. After 7 days, a 2-fold reduction in the tumor mass was observed; on the 14th day, a 4-fold reduction was detected, while on the 21st day, an 8-fold decrease was found. The tumor mass decreases without significant proliferation after 28 days due to the accumulation of paclitaxel in the tumor tissue, which reduces the tumor volume.

### 3.9. In Vivo Micelle Tracking

**Figure 11:**
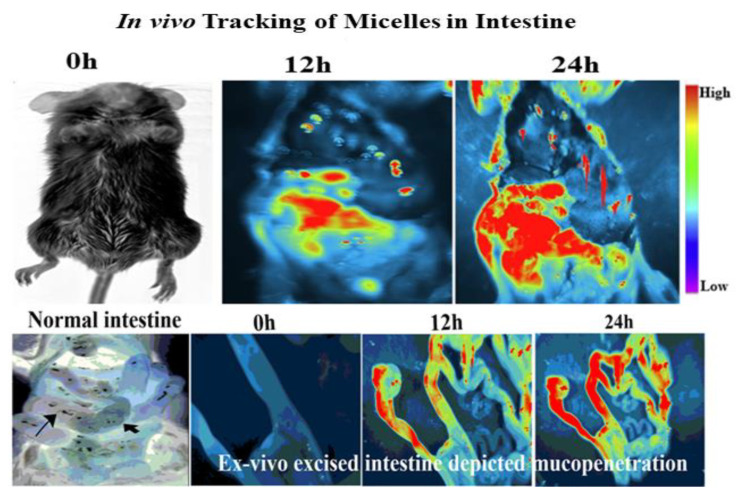
In vivo imaging of Balb C mice indicating the enhanced mucopenetration of the PT-R-Ms at different time intervals with a fluorescent dye (Nile red, 0.1% *w*/*w* dye loading). Ms: non-redox micelles, R-Ms: redox micelles, P-RMs: papain functionalized redox micelles, P-RMs: papain functionalized thiolated redox micelles.

To further demonstrate the distinct mucopermeating behavior of the PT-R-Ms, fluorotagged micelles were orally co-administered in mice, as shown in Figure 11. The PT-R-Ms were disseminated extensively throughout the intestine, but both the R-Ms and the Ms remained accumulated in the intestinal lumen. In vivo results further corroborated that the P-R-Ms retained their superiority in terms of mucus penetration and cellular internalization compared with the R-Ms and the Ms. This study suggests that enzyme grafted thiolated micelles can efficiently cross the mucus barrier by slicing mucus substructures ahead of them on their way to the epithelium. Our findings are in agreement with a previously published work in which negatively charged micelles repelled/did not adhere to polyanionic mucus gels [37].

### 3.10. In Vivo Pharmacokinetic Study

**Figure 12:**
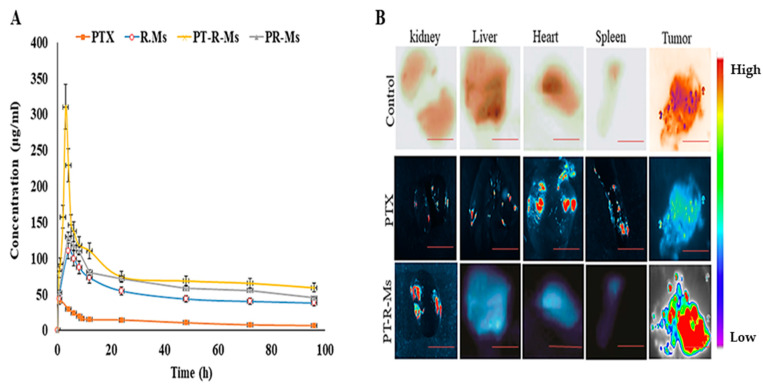
(**A**) Plasma concentration of PTX as a function of time after oral administration of PTX and PTX-loaded R-Ms, PR-Ms, and PT-R-Ms (Oral dose = 20 mg/kg). Blood samples were taken at different time intervals until 96 h and analyzed by the HPLC method for PTX quantification. The results are shown as mean ± SD (*n* = 3). (**B**) Biodistribution analysis of orally administered pure PTX and fluorescent probe labeled PT-R-Ms in mice organs along with the excised tumors. The scale bar is 20 µm.

The mean plasma concentration at different time intervals (1, 2, 4, 6, 12, 24, 48, 72, and 96 h) for PTX loaded R-Ms, PR-Ms, and PT-R-Ms is shown in Figure 12A, and the pharmacokinetic parameters are summarized in Table 4. It should be noted that the PTX-loaded PT-R-Ms attained the largest area under the curve (AUC). The value of AUC for PT-R-Ms was 10.8-fold higher than the area calculated for the pure PTX, while the area for P-R-Ms and R-Ms are 3.2- and 2.8-fold higher, respectively. It was observed that after oral administration, pure PTX reached the maximum concentration (Cmax) after 2 h and remained above the minimum effective concentration MEC (35 ng/mL) only for 2 h. On the other hand, R-Ms, P-R-Ms, and PT-R-Ms reached the MEC level after 15 min and remained within the therapeutic window for 96 h. Half-life (t_1/2_) of PT-R-Ms was 56.91 h, around 1.73-fold higher than that of the pure drug, while t_1/2_ of P-R-Ms and R-Ms was 44 and 38 h, respectively. Furthermore, a 3.25-fold increase in Cmax was found for the PT-R-Ms as compared to the pure PTX, while only a 2-fold and 1.13-fold increment was observed for P-R-M and R-Ms, respectively. The experimental data obtained herein show a 10.8-fold increase in the AUC 0-96 of PTX with PT-R-Ms as compared to PTX aqueous dispersion. These results indicate that PT-R-Ms redox micelles comprising PTX noticeably facilitated the oral absorption, owing to improved solubility and higher diffusion across the unstirred aqueous layer and the intestinal epithelial layers. The superior mucous penetration, P-gP efflux pump inhibition and pronounced apoptosis against tumor cells make the PT-R-Ms a more reliable drug delivery system to achieve higher absorption and bioavailability.

### 3.11. Biodistribution

Biodistribution of pure PTX showed significantly higher uptake in the liver due to non-specific clearance of the drug and extensive first-pass effect [38]. In the heart, spleen, and kidney, the biodistribution of PT-R-Ms was significantly lower while high fluorescent micelles were accumulated in the excised tumor, as depicted in Figure 12B. When the uptake of PT-R-Ms in the tumor was compared to other organs, significantly higher distribution was found due to the presence of papain enzyme that degrades the ECM present in the tumor tissue, thus allowing the penetration of micelles deep into the tumor tissue, hence hindering the absorption of the therapeutic entity and its specificity towards the target. This specific targeting ability is due to the presence of CD44 receptors on the tumor tissue; however, the results revealed that PT-R-Ms improved tumor targeting ability and reduced the distribution of PTX into vital organs. The highest retention of PT-R-Ms into the tumor tissue might be due to long circulation time [39].

### 3.12. Acute Oral Toxicity

The biosafety of PT-R-Ms was evaluated using rats. In vivo toxicity was examined in rats for 14 days with a single oral dose of 20 mg of PTX/kg. Normal saline was given to the control group while other groups were treated with either pure PTX dispersion, Ms, P-R-Ms, or PT-R-Ms. The physical parameters of this group, including behavior pattern, fur, skin changes, and so forth, were assessed. Mortality, tissue damage, or bodyweight change were not observed after 14 days. Essential parameters such as blood profile and serum analysis were performed on sacrificed mice after 14 days. To investigate the biocompatibility of the redox formulations, histopathological evaluation was performed for spleen, liver, heart, and kidney. Bodyweight ratio is an imperative element to estimate the in vivo toxicity of formulations.

**Figure 13:**
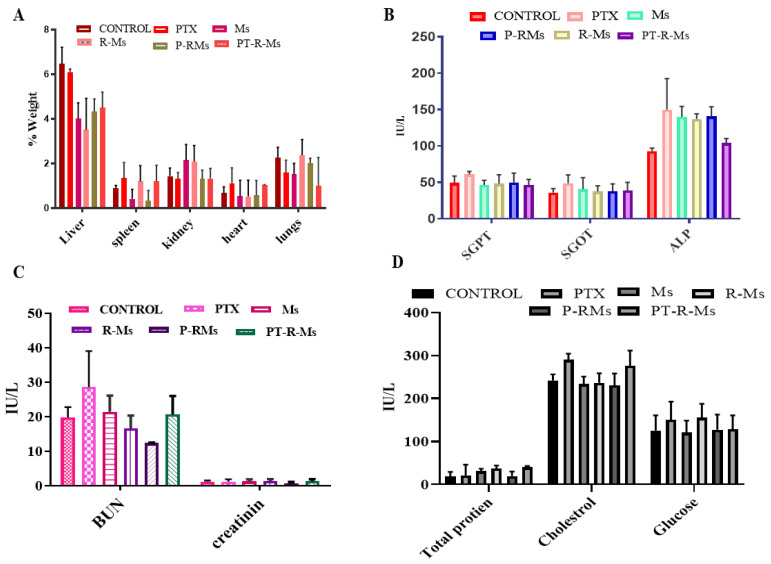
Serum biochemistry analysis of mice plasma after acute oral treatment with PTX, Ms, R-Ms, P-R-Ms, and PT-R-Ms with control to monitor changes on (**A**) body weight % (**B**) LFTs; (**C**) electrolytes; (**D**) total protein, cholesterol, and glucose content. Results are shown as mean ± SD (*n* = 3).

Alterations in body weight depict the level of stress, anxiety, and any abnormalities in response to the administration of drug formulations [40]. The results demonstrate that small changes took place in vital organs such as the liver, spleen, heart, and kidney (as shown in Figure 13A); however, these changes were found to be insignificant when compared with the control. Hence, it is confirmed that R-Ms, PR-Ms, and PT-R-Ms do not cause toxicity during the treatment period. Further analysis was performed on the plasma (as shown in Figure 13C), and no noticeable changes in bilirubin and creatinine ratio were detected following the administration of R-Ms, P-R-Ms, and PT-R-Ms compared to the control group (treated with saline), while pure PTX showed the highest levels; however, pure PTX increases the ratio of enzymes such as serum glutamic-pyruvic transaminase (SGPT) and serum glutamic-oxaloacetic transaminase (SGOT) because of its hepatotoxicity and tissue detrimental effect, since PTX metabolizes from the liver [36]. Nonetheless, PT-R-Ms micelles led to insignificant changes that fell within the normal range. PT-R-Ms consist of hyaluronic acid (HA) and lithocholic acid (LCA) that have a hepatoprotective effect [41]; hence no significant changes occurred in the levels of liver enzymes (SGPT and SGOT). Ms, R-Ms, P-R-Ms, and PT-R-Ms did not cause any degradation in the tissue level; HA possesses tissue regenerative ability, which accounts for the insignificant changes in the amounts of liver enzymes. Alkaline phosphatase (ALP) level decreased in the presence of PT-R-Ms, as shown in Figure 13B. The serum glucose, cholesterol, and total protein content were also estimated in the presence of Ms, R-Ms, P-R-Ms, and PT-R-Ms. As mentioned earlier, pure PTX increases the level of serum glucose, cholesterol, and protein, while HA-based redox formulations increase glucose and cholesterol intolerance by activating the apolipoproteins (A1) and high-density lipoprotein (HDL), and reduces protein content, so that it falls within normal limits, as shown in Figure 13.

### 3.13. Tissue Histology

**Figure 14:**
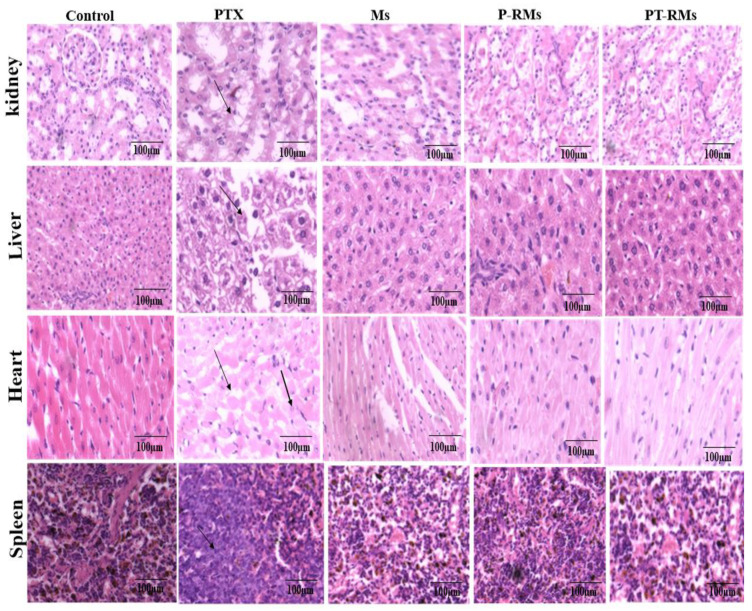
Histopathological changes of vital organs (spleen, heart, liver, and kidney) in H&E staining after 14 days. Pure PTX exhibited histological lesions in liver tissue, necrosis, and infiltration of inflammatory cells indicated with an arrow.

A comprehensive histopathological examination was performed to investigate the histological changes and explore potential side effects such as tissue damage, inflammation, degeneration, and lesion formation in vital organs such as liver, kidney, spleen, and heart after 14 days with PTX, RMs, PR-Ms, and PT-R-Ms (single oral dose of 20 mg/kg). No significant histological changes were observed in H&E-stained tissue sections of the liver, spleen, lung, or kidney (Figure 14) nor in the control (treated with normal saline) or the corresponding formulations with RMs, PR-Ms, and PT-R-Ms. No gross abnormalities were observed. Furthermore, no lesion, inflammation, or degeneration was observed with PT-R-Ms since it is considered non-toxic, while the histopathological changes observed in the liver tissue with the group treated with pure PTX indicated cytoplasmic degeneration, necrosis, hemorrhage, and infiltration of inflammatory cells. The histopathology of kidney tissue showed that the exposure of PTX caused distorted glomeruli, dilated tubules, edema exudate, mild necrosis, and infiltration of inflammatory cells. The spleen tissue was observed in the form of distorted lymphoid architecture, and the heart tissue showed lesions, myocarditis, and infiltration of inflammatory cells; however, formulations with Ms, PR-Ms, and PT-R-Ms hardly exhibited changes in the histology of vital organs.

## 4. Conclusions

We present an innovative strategy to develop mucopermeating papain functionalized thiolated redox micelles (PT-R-Ms) for site-specific delivery of PTX. The PT-R-Ms demonstrate higher penetration, enhanced efficacy, and improved oral bioavailability compared to conventional paclitaxel formulations. The current study reveals that the developed formulations can inhibit P-gP efflux pump with enhanced permeation and mucoadhesion. It also evidences substantial improvement in anticancer activity both in vitro and in vivo. Orally administered PT-R-Ms efficiently get through intestinal barriers and show significantly improved PTX bioavailability, hence increased biodistribution to target organs, i.e., kidneys, in comparison to pure PTX. In vivo results revealed the improved efficacy of polymeric nanomicelles against solid tumors. Overall, the results obtained herein demonstrate that PT-R-Ms hold great potential for targeting solid tumors safely and selectively. This study has demonstrated the proof of concept that mucopenetrating thiolated micelles would be worthful in tumor disease treatment in the future and provides evidence of success for this targeted delivery system. Future exploration could intend the mechanistic approach of PT-R-Ms for tumor-targeted delivery.

## Figures and Tables

**Table 1:** Nomenclature, chemical composition, and characteristics of the different polymeric micelles used in this work.

Formulation Code	Description	Chemical Composition	Synthesis Method	Size (nm)	EE %	DL %
**Ms**	Micelles	HA-F_127_	Probe sonication	105 ± 16	55 ± 14	50 ± 09
**R-Ms**	Redox micelles	HA-F_127_-SS LCA	Probe sonication	97 ± 12	67 ± 20	59 ± 04
**PR-Ms**	Papain functionalized redox micelles	Pap-HA-F_127_-SS-LCA	Probe sonication	95 ± 09	76 ± 21	79 ± 21
**PT-R-Ms**	Papain functionalized thiolated redox micelles	Pap-THA-F_127_-SS-LCA	Probe sonication	90 ± 07	82 ± 10	80 ± 14

Hyaluronic acid linked pluronic (HA-F_127_), hyaluronic acid-pluronic F_127_ disulfide-linked lithocholic acid (HA-F_127_-SS-LCA), papain grafted hyaluronic acid-pluronic F_127_ disulfide-linked lithocholic acid (Pap-HA-F_127_-SS-LCA), papain grafted thiolated hyaluronic acid-pluronic F127 disulfide-linked lithocholic acid.

**Table 2:** Drug release kinetic modeling demonstrates the potential drug release mechanisms.

Formulation Code	Zero Order		Higuchi Model		Korsmeyer–Peppas Model		Hixon Crowell Model	
	K_0_	R^2^	K_0_	R^2^	N	R^2^	K_0_	R^2^
**PTX**	21.01	0.987	40.117	0.912	0.701	0.912	0.190	0.891
**Ms**	0.573	0.511	6.109	0.312	0.232	0.894	0.002	0.234
**R-Ms**	0.713	0.639	7.019	0.412	0.214	0.612	0.004	0.213
**PR-Ms**	0.142	0.4532	8.321	0.514	0.491	0.131	0.001	0.413
**PT-R-Ms** **(no GSH)**	1.314	0.8123	10.123	0.645	0.309	0.928	0.001	0.315
**PT-R-Ms** **(20 mM GSH)**	1.936	0.5124	16.011	0.567	0.214	0.989	0.027	0.509

**Table 3:** Ex vivo permeation and efflux ratio of PTX in buffer solution, PTX with verapamil (PTX-Ver), Ms, R-Ms, P-R-Ms, and PT-R-Ms. Values are given as mean ± SD (*n* = 3).

Formulation	P_app_(A-B) (cm/s) × 10^−6^	IMPROVEMENT RATIO	P_app_(B-A) (cm/s) × 10^−6^	Improvement Ratio	Efflux Ratio B-A/A-B
**PTX in buffer**	0.43 ± 0.2		2.35 ± 0.2		5.46
**PTX + Ver**	0.71 ± 0.15	1.82	2.85 ± 0.2	1.43	4.01
**Ms**	0.78 ± 0.17	1.98	3.781 ± 0.2	1.49	4.84
**R-Ms**	1.98 ± 0.11	5.78	2.912 ± 0.01	1.31	1.471
**P-R-Ms**	2.17 ± 0.02	6.21	1.99 ± 0.01	1.17	0.917
**PT-R-Ms**	2.560 ± 0.1	7.89	1.76 ± 0.21	0.87	0.068

**Table 4:** Pharmacokinetic parameters and relative oral bioavailability obtained after oral administration of PTX suspension in deionized water, Ms, R-Ms, P-R-Ms, and PT-R-Ms to mice through oral gavage.

PK Parameters	Units	PTX	R-Ms	P-R-Ms	PT-R-Ms
**Cmax**	μg/mL	70.69 ± 0.14	80.09 ± 4.01	141.82 ± 1.05	228.91 ± 11.41
**Tmax**	h	4 ± 0.2	4 ± 0.2	3 ± 0.15	2 ± 0.1
**AUC 0-96**	μg/mL × h	4271.09 ± 8.4	4871.19 ± 8.9	6271.07 ± 10.7	7580.56 ± 11.8
**AUMC 0-96**	μg/mL × h^2^	442,433.01 ± 15.2	452,017.23 ± 15.1	640,134.31 ± 17.6	8,725,741 ± 18.90
***t*_1/2_**	h	32.87 ± 2.2	38.19 ± 2.3	44.01 ± 3.1	56.91 ± 4.8
**MRT 0-96**	h	54.88 ± 2.7	52.697 ± 2.4	57.01 ± 2.6	67.074 ± 3.3
**F**	%	7.5	9.91	10.17	17.12

## Data Availability

The data are available on reasonable request from the corresponding author.
